# Supplementary figures and images for: Response of bitter and sweet Chenopodium quinoa varieties to cucumber mosaic virus: Transcriptome and small RNASeq perspective
Source: PLoS One. 2021 Feb 23;16(2):e0244364. doi: 10.1371/journal.pone.0244364 (PMC7901783; doi:10.1371/journal.pone.0244364)

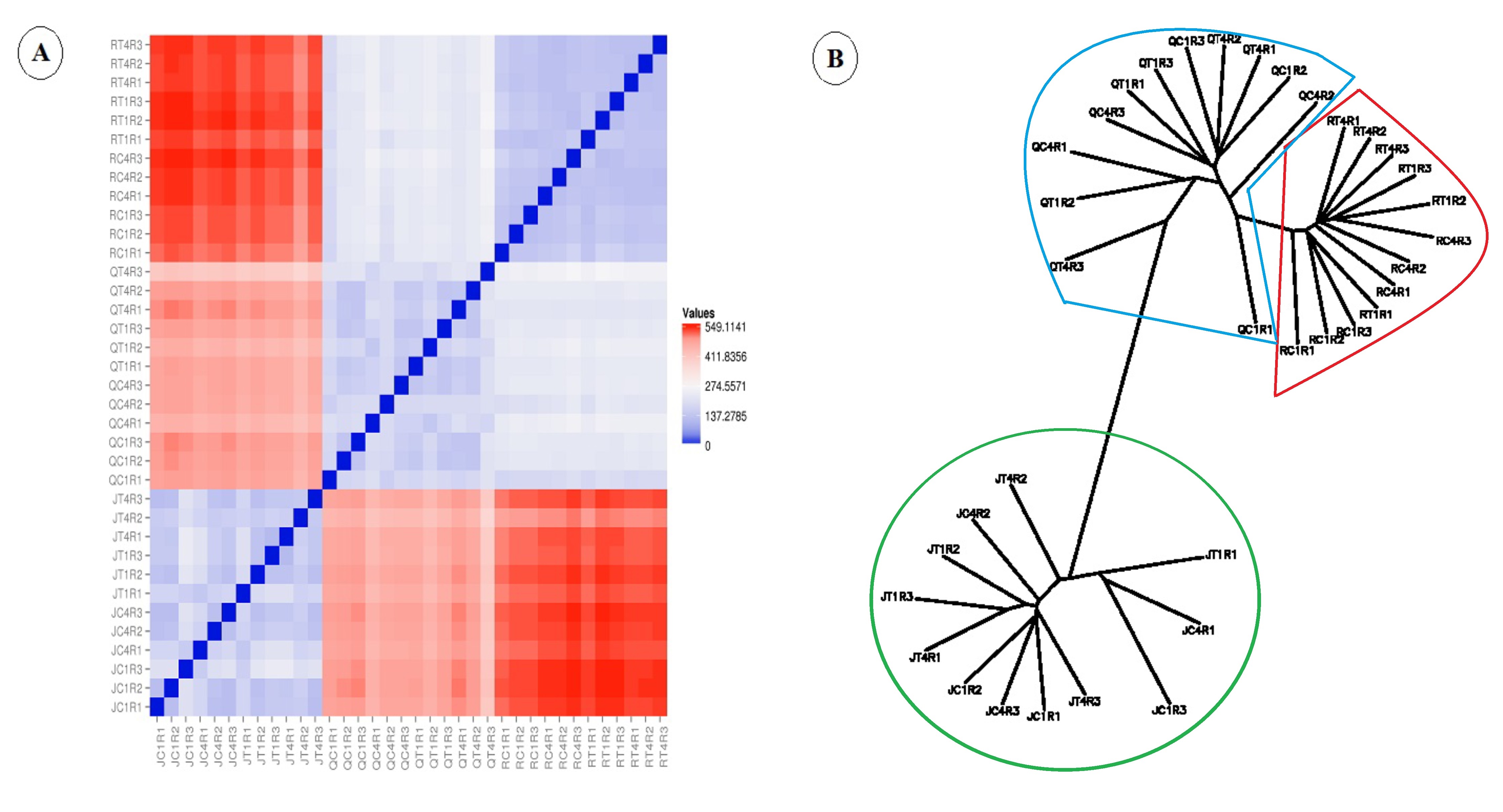

Supplement: S1 Fig — (A) Distance matrix within and among samples. In color legend, the blue representing the closest distance between samples and the red represents farther distance between samples. (B) Unrooted phylogenetic tree of quinoa varieties with their biological replicates. J, Q, and R are initials of the varieties, 1,4 are days post inoculation, and respective biological replicates represented by either R1, R2, or R3 symbols. There was close relationship among the individual samples of each variety, however they were not identical (S1A Fig). Higher similarity between ‘Red Head’ and ‘QQ74’ than ‘Jessie’ which is genetically distant are highlighted in the phylogram (S1B Fig). (JPG) [file pone.0244364.s001.jpg]

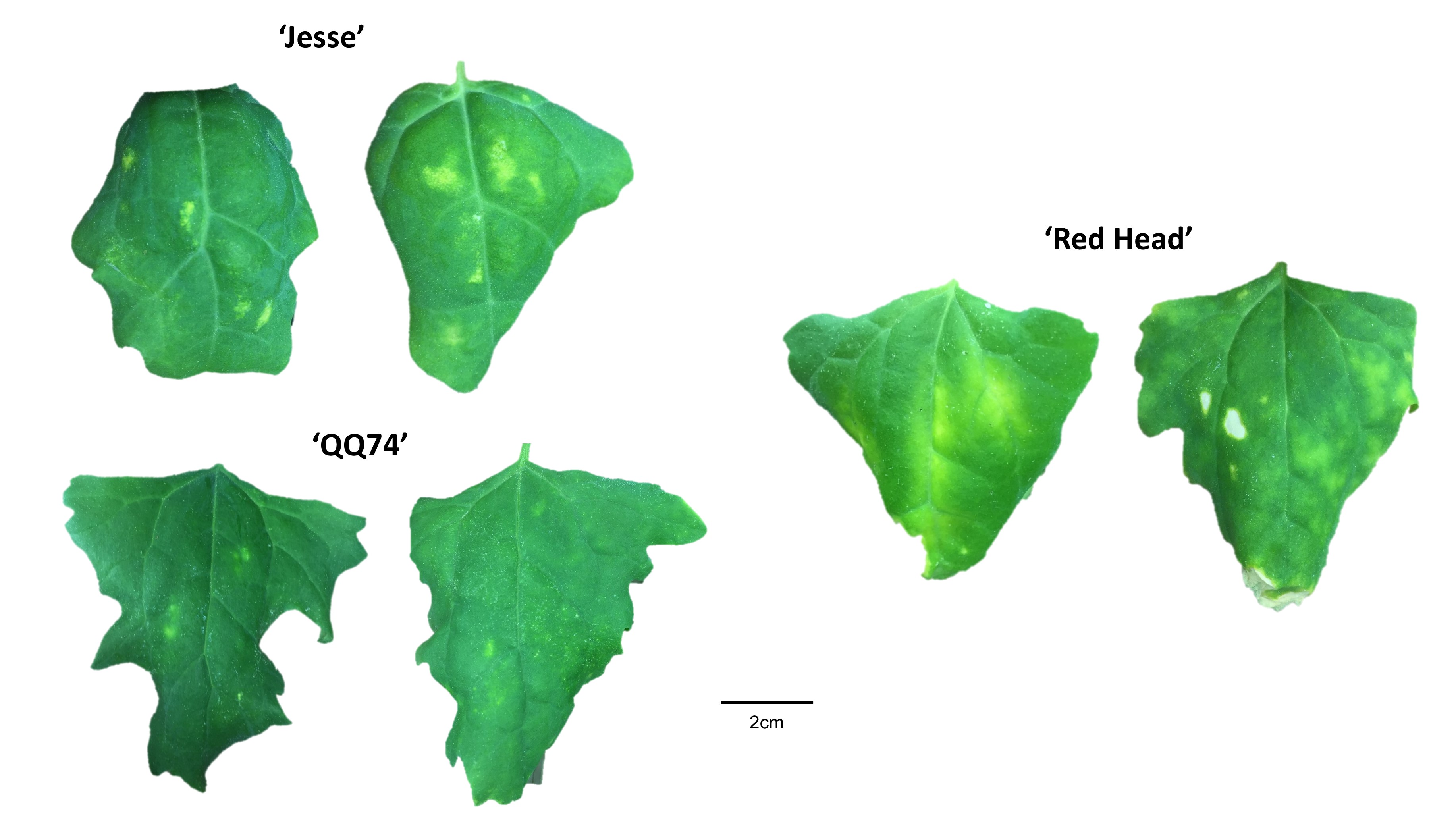

Supplement: S2 Fig — (JPG) [file pone.0244364.s002.jpg]

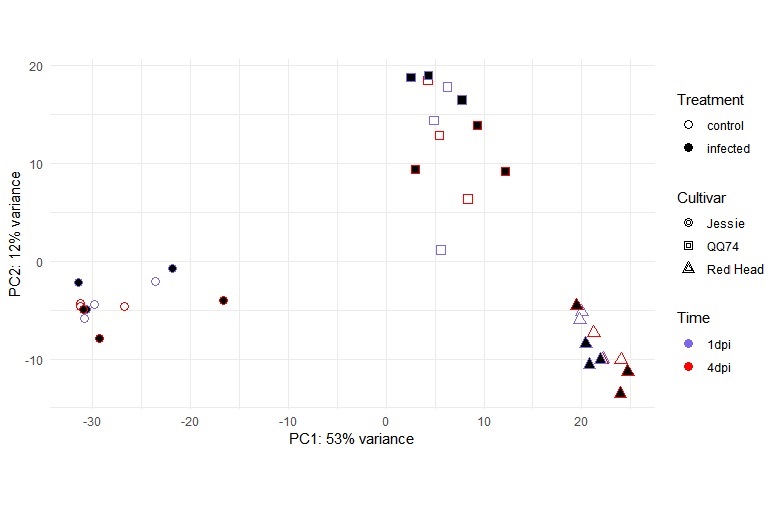

Supplement: S3 Fig — The treatments are infected quinoa inoculated with CMV or control as mock-inoculated samples; in varieties, ‘Jessie’ is low saponin (sweet variety), ‘QQ74’ is the medium saponin (sweet variety), and ‘Red Head’ is high saponin (bitter variety); and the time (1 or 4) is the harvesting time post CMV inoculation (dpi). (JPG) [file pone.0244364.s003.jpg]

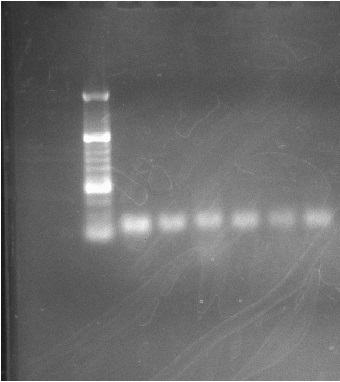

Supplement: S6 Fig — (JPG) [file pone.0244364.s006.jpg]

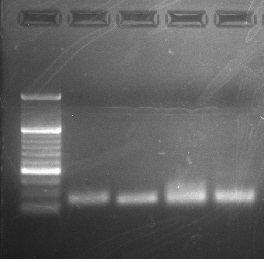

Supplement: S7 Fig — (JPG) [file pone.0244364.s007.jpg]

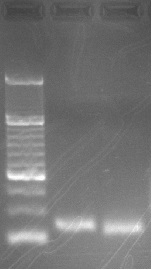

Supplement: S8 Fig — (JPG) [file pone.0244364.s008.jpg]

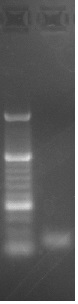

Supplement: S9 Fig — (JPG) [file pone.0244364.s009.jpg]

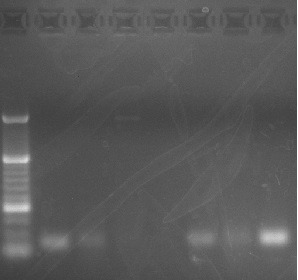

Supplement: S10 Fig — (JPG) [file pone.0244364.s010.jpg]

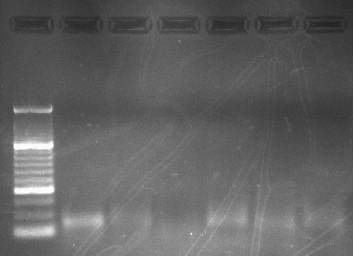

Supplement: S11 Fig — (JPG) [file pone.0244364.s011.jpg]
